# Efficacy of Osteopathic Manipulative Treatment in Creating a Difference in Pain Levels for Patients With Localized Joint Pain: A Meta-Analysis of Randomized Controlled Trials

**DOI:** 10.7759/cureus.82724

**Published:** 2025-04-21

**Authors:** Jason S DeFrancisis, Benjamin M Vinarski, Melany Abreu, Jalal Ibrahim, Robert Hostoffer

**Affiliations:** 1 Orthopedic Surgery, Lake Erie College of Osteopathic Medicine, Bradenton, USA; 2 Internal Medicine, Lake Erie College of Osteopathic Medicine, Bradenton, USA; 3 Psychiatry, Lake Erie College of Osteopathic Medicine, Bradenton, USA; 4 Dermatology, Lake Erie College of Osteopathic Medicine, Bradenton, USA; 5 Allergy and Immunology, Case Western Reserve University, Cleveland, USA

**Keywords:** elbow joint pain, joint pain, knee joint pain, orthopedic intervention, orthopedics, osteopathic manipulative medicine, osteopathic manipulative treatment (omt), shoulder joint pain

## Abstract

Osteopathic manipulative treatment (OMT) is a therapy utilized by osteopathic physicians in diverse clinical environments. Among the many uses, OMT may be used in attempts to relieve pain in patients. Joint pain is among the most common causes of pain, with millions worldwide suffering from joint pain. This meta-analysis examines the efficacy of OMT in creating a difference in pain levels for patients with localized joint pain. This meta-analysis, comprising three studies and 187 patients, found a mean difference in pain between OMT and non-OMT groups using the common effects model to be -3.09 (-3.57; -2.61) with p<0.0001, suggesting that OMT resulted in a significant pain difference in those with joint pain. However, the random effects model showed a mean difference of -1.80 (-7.31; 3.78), which was not statistically significant. Furthermore, the meta-analysis at hand had a heterogeneity measure, known as I^2^, at 96%, suggesting that the results should be interpreted with caution. Further analysis showed that shoulder pain reduction had a significant influence on the overall study results, and sub-analysis showed that OMT can result in a significant reduction in pain in patients with localized shoulder pain, with a mean difference of -4.28 (-4.86; -3.70) and p<0.0001. Overall, given the discrepancy in results between the common effects model, random effects model, and high heterogeneity, a conclusive statement on whether OMT can cause a significant difference in joint pain cannot be made. Further research with standardized treatment lengths, larger sample sizes, and more homogeneous patient populations is needed to accurately assess the true impact.

## Introduction and background

Osteopathic manipulative treatment (OMT) was established on the principle that the body is a dynamic unit where structure and function are deeply connected in both health and disease [1]. As a method of treatment, OMT is used to accentuate the body's natural ability to recover from displacements, derangements, and consequent disease, thereby returning to its normal form and function [2]. At its core, OMT is grounded in four key principles: the body is a dynamic unit, the body has self-regulatory mechanisms that promote healing, structure and function are interrelated at all levels, and rational treatment is based on these fundamentals [3]. The application of OMT is often centered around the diagnosis and treatment of somatic dysfunctions, which are restrictions that can occur in bones, joints, muscles, and fascia [4]. These restrictions affect blood supply, lymphatic flow, and nerve function [5]. However, of the various specific pathologies that OMT can address, acute and/or chronic pain happens to be among the most common causes [6]. OMT serves as a valuable non-pharmacological treatment approach to managing pain, offering a promising alternative or complement to traditional management [7]. OMT’s effectiveness in treating chronic joint pain in particular has been demonstrated through prior research and, in turn, has the potential to become a mainstay conservative treatment measure [8].

The integration of OMT in the management of joint pain is warranted, as joint pain is one of the leading causes of chronic disability in the United States [9]. It is becoming an increasingly exhaustive burden on the public, with past estimates of nearly 40% suffering from joint pain daily [10]. Localized joint pain may be caused by inflammation of the joint capsule, arthritis of the synovial lining, or traumatic injury disrupting the architecture of the joint itself [11]. Many patients also report reductions in functional ability and seek the most optimal care for their condition [9]. Some joints are affected more frequently than others. The knee, for example, is often mentioned as a primary source of pain and chronic disability, particularly due to osteoarthritis [12]. In addition, shoulder pain is also widespread, with a lifetime prevalence of up to 70%, often caused by rotator cuff injuries, which can lead to chronic discomfort and ongoing complaints from patients [13]. While less common, elbow pain still accounts for around 35% of joint complaints, often related to rheumatoid arthritis or lateral epicondylitis [14].

Exploring OMT as an adjunct modality to the current treatment for joint pain is an area of interest given this exhaustive burden for patients. Current treatment modalities often utilize adjacent therapies consisting of pharmacologic and physical approaches prior to invasive or minimally invasive techniques, but it comes with a cost [9]. The annual cost of pain and pain management in the United States was estimated to be greater than the annual costs of heart disease, cancer, and diabetes, ranging from 560 to 635 billion dollars in 2010 [15]. OMT can therefore serve as an alternative or adjunct therapy because it has led to reduced outcomes requiring interventional therapies, radiology services, and fewer opioid prescriptions [16].

This has led us to investigate optimal accessory treatment modalities and pose the question: does OMT cause a significant difference in pain than non-OMT in patients with localized joint pathologies? Specifically, this study focuses on three distinct and isolated joint pathologies - shoulder, elbow, and knee pain - and cross-examines the effectiveness of OMT in reducing pain in each area. We hypothesize that OMT will produce a statistically significant difference in joint pain when compared to non-OMT modalities.

## Review

Materials and methods

Research Question

Does the use of OMT cause a significant difference in pain levels between adult patients who received OMT versus those who received non-OMT for localized joint pain?

Inclusion Criteria

The study population included adult males and females aged 18 years or older who suffered from joint pain in the shoulder, elbow, or knee. The study design included randomized controlled trials. The intervention being measured was the use of OMT versus non-OMT. The primary outcome of pain level after treatment (either OMT or non-OMT) was assessed using a 10-cm visual analog scale (VAS). Only studies published in English were considered.

Exclusion Criteria

Non-relevant studies unrelated to OMT for joint pain were excluded. Excluded study designs consisted of non-randomized controlled trials: case reports, cross-sectional studies, systematic reviews, and prospective or retrospective cohorts. Studies available only in non-English were excluded, those published before 2010 were not considered, and those that did not report pain on the VAS were omitted.

Search Strategy

**Figure 1:**
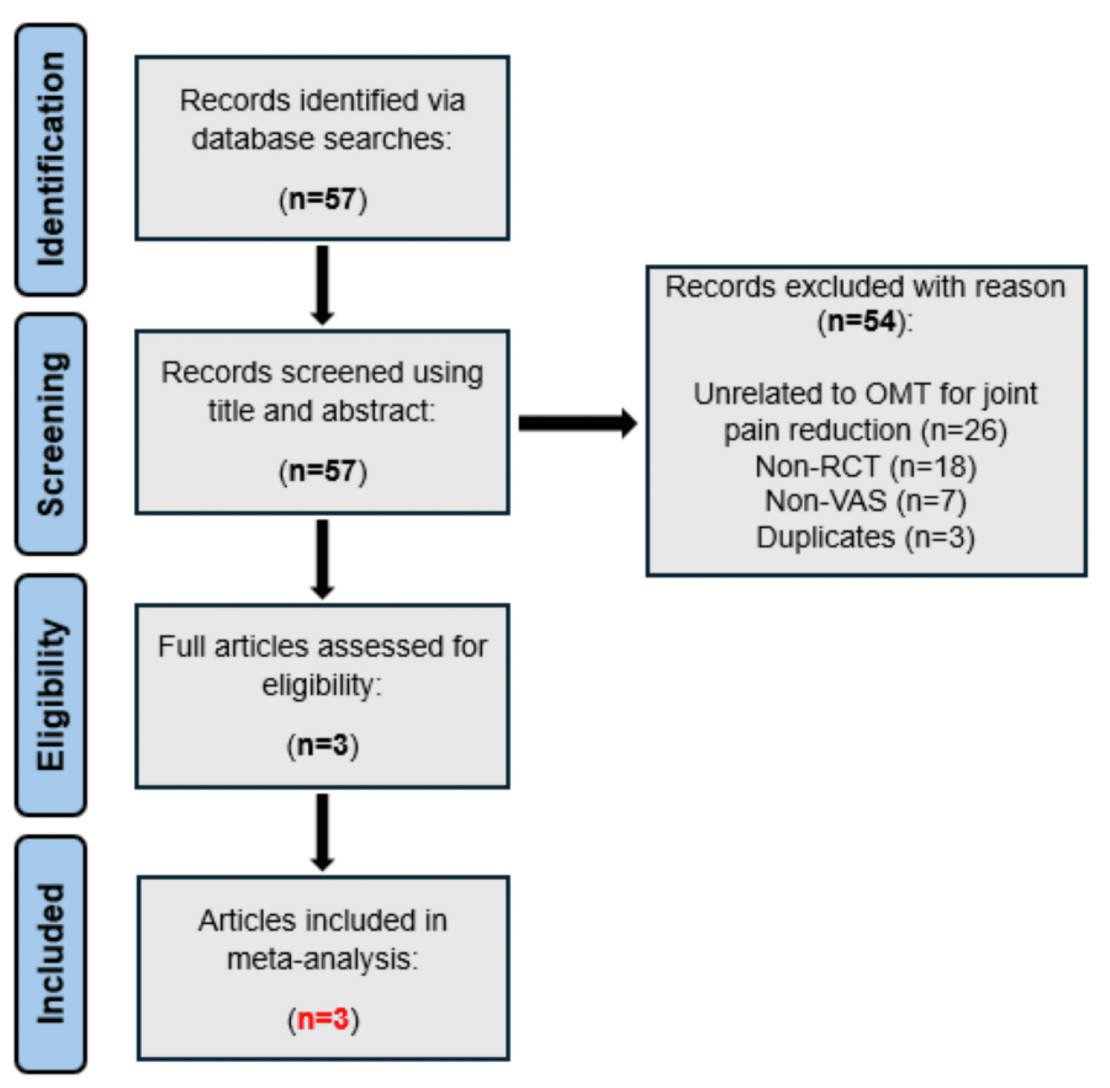
A PRISMA flow chart displaying the literature search and selection process n, number; non-RCT, non-randomized controlled trials; non-VAS, non-visual analog scale; OMT, osteopathic manipulative treatment; PRISMA, Preferred Reporting Items for Systematic Reviews and Meta-Analysis

The Preferred Reporting Items for Systematic Reviews and Meta-Analyses (PRISMA) criterion (Figure 1) was implemented for the review [17]. An extensive electronic database search was carried out on titles up to December 2024 in the following databases: PubMed, Elsevier, ResearchGate, Google Scholar, AOA Osteopathic Research Web, AOA Osteopathic Research Database, American Academy of Osteopathy Journal, and OSTMED.DR. Search terms included “joint pain”, “knee pain”, “shoulder pain”, “elbow pain”, “pain relief”, “randomized controlled trial”, “OMT”, “osteopathic manipulative treatment”, “osteopathic treatment”. Several search strings were employed to identify relevant studies: “Osteopathic Manipulative Treatment AND Joint Pain AND Randomized Controlled Trials”, “OMT Techniques AND Pain Relief AND Visual Analog Scale”, and “Heterogeneity AND Meta-Analysis AND Osteopathic Manipulative Treatment”. Boolean operators (AND/OR) were assembled to combine terms, and selective filters for study design (randomized controlled trials), publication date (2010-2024), and language (English) were used to concentrate results.

Data Sources

Manual searches of pertinent systematic reviews and meta-analyses were additionally performed to identify studies not captured in our database searches.

Study Selection

Duplicates, non-randomized controlled trials, and studies unrelated to OMT and joint pain were not included in the statistical analysis. There were no disagreements between the two reviewers (J.D. and B.V.).

Data Extraction

Data related to study design, sample size, standard deviation, confidence intervals, and average pain rating post-treatment on a 10-cm VAS were extracted from each study. Conversion to 10-cm VAS was utilized for studies that reported such data on a 100-mm VAS.

Quality Assessment

Only randomized controlled trials were included in an attempt to minimize bias within each study. Bias was assessed using the NIH Study Quality Assessment Tool and statistical analysis [18].

Statistical Analysis

All analyses and graphs were produced utilizing RStudio (v4.4.1, R Core Team, 2024) and the meta package version 7.0-0. A forest plot was constructed to illustrate the mean differences and corresponding confidence intervals for each study, thereby providing an overall effect estimate. The random-effects model, incorporating 95% confidence intervals, was utilized to assess heterogeneity among studies. Heterogeneity was evaluated using Cochran’s Q-test, the I² statistic, and Tau² to analyze variability across studies.

Assessment of Results

Our meta-analysis calculated both the common effects model and the random effects model. In the subsequent discussion, both models will be discussed. It should be noted that when interpreting studies with a high heterogeneity, it is generally better to use the random effect model for interpretation [19]. However, when there are too few studies to obtain an accurate estimate of between-studies variance, the common effects model may be used [20]. Due to the aforementioned reasons, we decided to include a discussion of both models to further explore all the results of this study.

Publication Bias

The NIH Study Quality Assessment Tool was implemented for each study to determine the bias associated with the included randomized controlled trials (Table 2) [18]. Two reviewers (J.D. and B.V.) were assigned to conduct the quality assessment independently. In the event of a disagreement upon responses to the provided questions, the two researchers engaged in discussion until a consensus was reached. The included studies received one point for each “yes” response and zero points for every “no” response to the questions provided by the NIH Study Quality Assessment Tool. An article was deemed “poor” if it received 0-4 points total, “fair” if it received 5-9 points total, and “good” if it received 10-14 points total.

Results

*Literature Search*

The total number of studies found was 57. After the removal of duplicates and unrelated articles to OMT for joint pain, three articles were evaluated and incorporated in this meta-analysis (Figure 1) [21-23].

Characteristics of Studies Included

Three studies were included in this meta-analysis [21-23]. The characteristics of each study are summarized in Table 1.

**Table 1:** Literature search results included in statistical analysis HVLA, high-velocity and low amplitude; MET, muscle energy technique; MR, myofascial release; RCT, randomized controlled trial; STT, soft tissue technique; VAS, visual analog scale

Study	Total Sample Size (N)	OMT Group Size (N)	Non-OMT Group Size (N)	Design	Osteopathic Manipulative Treatment	Anatomic Region	Pain Scale Measured
Altinbilek et al., 2018 [21]	85	44	41	RCT	Mobilization and compression, pedal pump	Knee: patellofemoral joint, tibiofemoral joint	VAS
Schwerla et al., 2020 [22]	70	36	34	RCT	Custom-tailored osteopathic treatment	Shoulder: glenohumeral joint, acromioclavicular joint, sternoclavicular joint	VAS
Altadonna et al., 2022 [23]	32	15	17	RCT	STT, MR, MET, HVLA	Elbow: lateral epicondyle	VAS

Risk-of-Bias Assessment

The risk of bias was assessed using the NIH Study Quality Assessment Tool [18]. The risk of bias in Altinbilek et al. was “fair” , the risk of bias in Schwerla et al. was “good” , and the risk of bias in Altadonna et al. was “fair” (Table 2) [21-23].

**Table 2:** Risk-of-bias quality assessment of controlled intervention studies Items 1-14 represent the 14 criteria questions used in the NIH Quality Assessment of Controlled Invention Studies. Each question is answered with a "yes" or "no" answer [18]. Based on the bias assessment tool, the NIH Quality Assessment of Controlled Intervention Studies, which measures methodological quality of randomized controlled trials [18]. F, fair; G, good; N, no; Y, yes

Study	Bias	1	2	3	4	5	6	7	8	9	10	11	12	13	14
Altinbilek et al., 2018 [21]	F	Y	Y	N	N	Y	Y	Y	Y	Y	Y	Y	N	N	N
Schwerla et al., 2020 [22]	G	Y	Y	Y	N	N	Y	Y	Y	Y	N	Y	N	Y	Y
Altadonna et al., 2022 [23]	F	Y	Y	N	N	N	Y	Y	Y	Y	N	Y	Y	N	N

**Figure 2:**
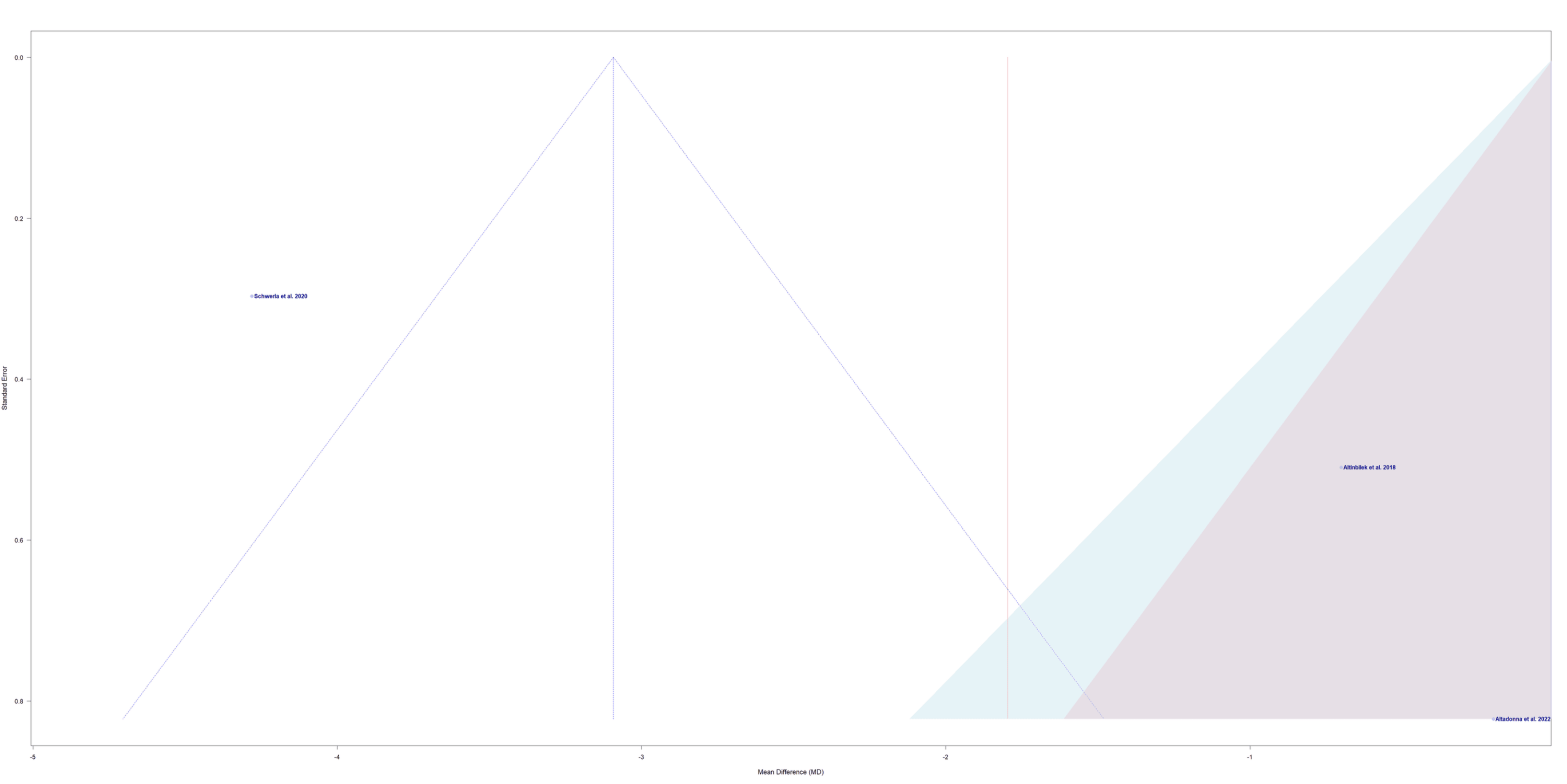
A funnel of publication bias for Altinbilek et al., Schwerla et al., and Altadonna et al. Studies included: Altinbilek et al., Schwerla et al., and Altadonna et al. [21-23].

The funnel plot in Figure 2 assesses the risk of bias across the individual selected studies [21-23]. Schwerla et al.’s study, the study closest to the midline, has the lowest risk of bias or variability (Figure 2) [22]. Altadonna et al.’s study was the furthest away from the midline, representing the highest risk of bias or variability (Figure 2) [23]. The lowest data point on the plot, which is Altadonna et al.’s study, represents the study with the smallest effect size (Figure 2) [23].

Findings

**Figure 3:**
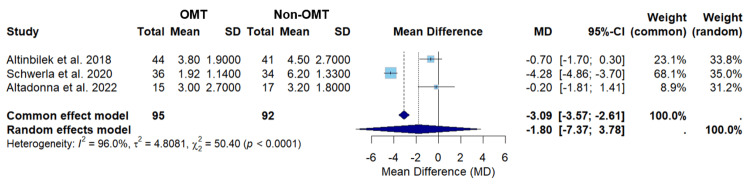
A forest plot of pain assessment using a visual analog scale compares OMT vs non-OMT for all studies included. Studies included: Altinbilek et al., Schwerla et al., and Altadonna et al. [21-23] CI, confidence interval; MD, mean difference; OMT, osteopathic manipulative treatment; SD, standard deviation

The results for common and random effect models in this manuscript can be found in Figure 3.

The meta-analysis consists of three randomized controlled trials (Figures 1, 3). The total number of participants was 187, of which 95 participants received OMT and 92 did not receive OMT. The overall effect size for all the studies with the common effects model was found to have a mean difference of -3.09 (-3.57; -2.61), with p<0.0001 (Figure 3). The overall effect size for all the studies with the random effects model was found to have a mean difference of -1.80 (-7.37; 3.78) (Figure 3). The common effects model suggests OMT had a statistically significant difference in pain levels between patients who received OMT versus those who received non-OMT for localized joint pain; however, the random effects model did not suggest that OMT had a statistically significant difference (Figure 3). The overall heterogeneity, I2 value, of 96% suggests considerable heterogeneity and indicates that the hypothesis of no heterogeneity may be rejected (Figure 3). A high heterogeneity reflects a substantial degree of variability among the studies analyzed [24]. Schwerla et al. had the greatest effect size, indicating that the magnitude of the difference in pain due to OMT was the highest, with a mean difference of -4.28 (-4.86; -3.70) (Figure 3) [22]. The effect size of Schwerla et al. was noted to be extensively different compared to the effect size of Altinilek et al. and Altadonna et al, in which the latter two were more similar (Figure 3) [21-23]. The box size is proportional to its weight, with Schwerla et al. having the highest weight in both the common (68.1%) and random (35.0%) effect models (Figure 3) [22]. Furthermore, Schwerla et al. had the most precision with the smallest confidence interval range (-4.86; -3.70) (Figure 3) [22]. On the other hand, Altadonna et al. had the lowest weight in both the common (8.9%) and random (31.2%) effects model (Figure 3) [23]. Likewise, Altadonna et al. had the least precision with the greatest confidence interval range (-1.81; 1.41) (Figure 3) [23].

**Figure 4:**
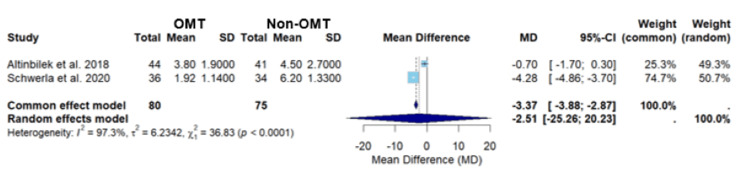
A forest plot of pain assessment using a visual analog scale comparing OMT vs non-OMT for Altinbilek et al. and Schwerla et al. Studies included: Altinbilek et al. and Schwerla et al. [21,22] CI, confidence interval; MD, mean difference; OMT, osteopathic manipulative treatment; SD, standard deviation

In Figure 4, the meta-analysis of studies by Altinbilek et al. and Schwerla et al. yielded mean differences for the common and random effects models of -3.37 (-3.88; -2.87) and -2.51 (-25.26; 20.23), respectively (Figure 4) [21,22]. The common effects model demonstrated statistical significance (p<0.0001), suggesting that OMT had a statistically significant difference in pain levels between patients who received OMT versus those who received non-OMT for localized joint pain (Figure 4). However, the wide confidence interval in the random effects model indicates substantial uncertainty in the estimate (Figure 4). The heterogeneity of this analysis was high, I2 = 97.3%, which is slightly higher than the heterogeneity seen when including all three studies, I2 = 96% (Figures 3, 4). The marginal increase in heterogeneity suggests there is slightly less consistency between the studies by Altinbilek et al. and Schwerla et al. [21,22,24]. The high heterogeneity warrants caution when interpreting the data [24].

**Figure 5:**
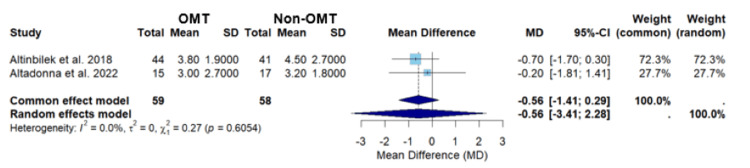
A forest plot of pain assessment using a visual analog scale comparing OMT vs non-OMT for Altinbilek et al. and Altadonna et al. Studies included: Altinbilek et al. and Altadonna et al. [21,23] CI, confidence interval; MD, mean difference; OMT, osteopathic manipulative treatment; SD, standard deviation

In Figure 5, the meta-analysis of studies conducted by Altinbilek et al. and Altadonna et al. yielded mean differences for the common and random effects models of -0.56 (-1.41; 0.29) and -0.56 (-3.41; 2.28), respectively (Figure 5) [21,23]. Neither the common effects nor the random effects models demonstrated statistical significance (p=0.6054), suggesting that OMT did not show a statistically significant difference in pain levels between patients who received OMT versus those who received non-OMT for localized joint pain (Figure 5). The analysis demonstrated no heterogeneity, I2 = 0.0%, indicating consistency in the treatment effects across the studies by Altinkbilek et al. and Altadonna et al. (Figure 5). [21,23,24]. The lack of heterogeneity warrants caution when interpreting the data [24].

**Figure 6:**
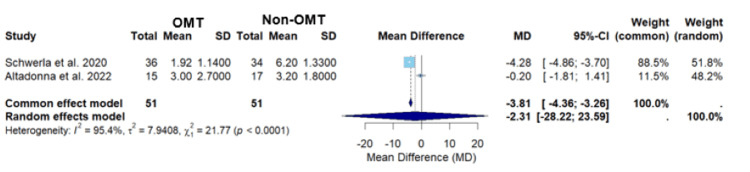
A forest plot of pain assessment using a visual analog scale comparing OMT vs non-OMT for Schwerla et al. and Altadonna et al. Studies included: Schwerla et al. and Altadonna et al. [22,23] CI, confidence interval; MD, mean difference; OMT, osteopathic manipulative treatment; SD, standard deviation

In Figure 6, the meta-analysis of the studies conducted by Schwerla et al. and Altadonna et al. yielded mean differences for the common and random effects models of -3.81 (-4.36; -3.26) and -2.31 (-28.22; 23.59), respectively (Figure 6) [22,23]. The common effects model demonstrated statistical significance (p<0.0001), suggesting that OMT had a statistically significant difference in pain levels between patients who received OMT versus those who received non-OMT for localized joint pain (Figure 6). However, the wide confidence interval in the random effects model indicates substantial uncertainty in the estimate (Figure 6). The heterogeneity of this analysis was high, I2 = 95.4%, which is slightly lower than the heterogeneity seen when including all three studies, I2 = 96% (Figures 3, 6). The marginal reduction in heterogeneity suggests that there is slightly more consistency between the studies by Schwerla et al. and Altadonna et al. [22-24]. The high heterogeneity warrants caution when interpreting the data [24].

**Figure 7:**
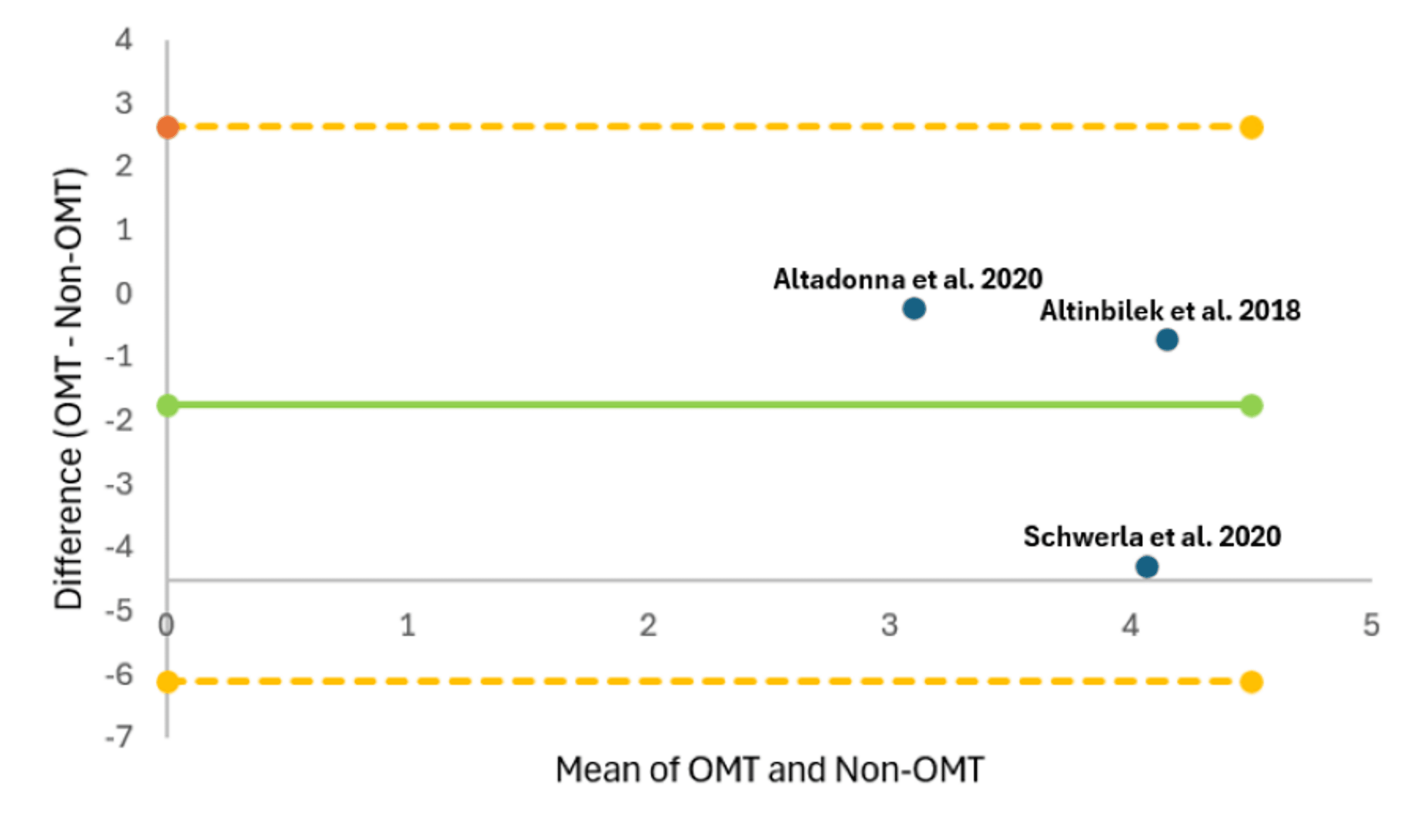
The Bland-Altman plot for pain measured using a visual analog scale after OMT versus non-OMT. The upper orange dashed line indicates the upper limit of agreement, the lower orange dashed line indicates the lower limit of agreement, the middle green solid line indicates bias, and dots labeled with each study indicate the difference between OMT versus non-OMT [21-23]. OMT, osteopathic manipulative treatment

The Bland-Altman plot visualizes the difference between VAS scores after OMT and non-OMT interventions against the average VAS scores for the included studies (Figure 7). The Bland-Altman plot reveals that the mean difference falls below zero (Figure 7). The data points for the three studies included all lie within the calculated limits of agreement, and thus this observation must be interpreted with caution due to the limited number of included studies [25]. On average, OMT may lead to lower VAS scores compared to non-OMT, as suggested by the negative bias [25]. The low sample size limits the ability to make a definitive conclusion regarding the agreement between the two measurement methods [25].

Discussion

The meta-analyses of three studies assessed if OMT caused a significant decrease in pain levels in patients with localized joint pain compared to non-OMT treatments. Throughout the studies assessed, various OMT techniques were employed, including mobilization and compression, pedal pump, soft tissue techniques, myofascial release, muscle energy techniques, and high-velocity and low amplitude, along with custom tailored-osteopathic treatment (Table 1). Joints treated in the analysis included the shoulder (glenohumeral joint, acromioclavicular joint, sternoclavicular joint), knee (patellofemoral joint and tibiofemoral joint), and elbow (lateral epicondyle) (Table 1). The primary outcome was to assess patients' joint pain levels using a VAS.

The findings from our meta-analysis indicate that the evidence regarding the effectiveness of OMT in reducing pain across all joints remains inconclusive. However, a significant reduction in pain was observed in patients with localized shoulder pain. Taking into account all three studies, with a total of 187 participants (95 OMT and 92 non-OMT), there was an average VAS pain score of 3.09 points lower in the OMT group compared to the non-OMT group (p<0.0001) using the common effects model, which was statistically significant (Figure 3). On the other hand, the random effects model showed that there was an average VAS pain score of 1.80 points lower in the OMT group compared to the non-OMT group, which was found to not be statistically significant (Figure 3). Despite this finding, a large heterogeneity was recognized (I2 = 96%). A high heterogeneity reflects a substantial degree of variability among the studies analyzed [24]. The high heterogeneity observed in this study may be attributed to the inclusion of multiple joints, the variation in OMT techniques applied, and the differing treatment frequencies. Therefore, the data presented may not be similar enough to create clinical interpretations and applications. However, a previous study conducted by Thorlund et al. has found that meta-analyses with a limited number of studies and small sample sizes must be interpreted cautiously, as they tend to possess large variations in I2 regardless of true heterogeneity [24]. The study identified that heterogeneity is likely to fluctuate considerably in meta-analyses with less than roughly 500 events and 15 trials [24]. The study also concludes that conference intervals for I2 are valuable in reflecting the uncertainty associated with estimating a high I2 [24]. Unfortunately, the meta-analysis software package utilized in our meta-analysis did not include the heterogeneity confidence interval. Due to the stated reasons, we believe that our heterogeneity value may have been overestimated when comparing all three studies.

Overall, the conclusion from this study should be taken with caution as only three randomized controlled trials were included. In addition, the results from the meta-analysis have some concerns due to the weight carried by Schwerla et al.’s study [22]. In the analysis conducted with all three studies, Schwerla et al.’s study is the only study with statistically significant results in pain reduction (Figure 3) [21-23]. In addition, Schwerla et al.’s study has a weight of 68.1% in the common effects model and 35.0% in the random effects model (Figure 3) [22]. The studies conducted by Altinbilek et al. and Altadonna et al. have confidence intervals that cross zero, indicating non-significant results [21,23,26]. Given these findings, the overall results of the meta-analysis are heavily influenced by Schwerla et al.’s study [22]. The considerable effect of OMT on shoulder pain reduction may offset the milder effects of OMT in other regions. In addition, since Schwerla et al.’s study is the major study showing a significant difference, the true effect of OMT on specific regions may be obscured [22]. With this being stated, the conclusion can be made that the impact of OMT varies based on the joint treated and had the most impactful pain reduction in the shoulder as this was the joint that Schwerla et al.’s study assessed (Table 2) [22].

The above statement is further supported through the sub-analysis comparing the studies by Schwerla et al. versus Altinbilek et al. and the sub-analysis of the studies by Schwerla et al. versus Altadonna et al. (Figures 4, 6) [21-23]. The comparison of the studies by Schwerla et al. and Altinbilek et al. demonstrated a statistically significant average VAS pain score (p<0.0001) using the common effects model, but the random effects model showed no statistically significant finding (Figure 4) [21,22]. Similarly, the sub-analysis of the studies by Schwerla et al. and Altadonna et al. displayed a statistically significant average VAS pain score (p<0.0001) using the common effects model; however, the random effects model once again showed that there was no statistically significant finding (Figure 6) [21,23]. Given the significant weight of Schwerla et al.’s study in both the sub-analysis and across the common and random effects models, it can be concluded that this study had a substantial impact on the results (Figure 4, 6) [21-23]. A secondary conclusion that can be made is that pain reduction not only varies with the region treated but also with the techniques used. As shown in Schwerla et al.’s study, tailored OMT techniques seem to be more effective than more standard techniques (Table 2) [22].

The sub-analysis of the studies by Altinbilek et al. and Altadonna et al. showed no statistical significance in the effect of OMT compared to non-OMT in pain levels; this applied to both the common or random effects model (p = 0.6054) (Figure 5) [21,22]. Interestingly, this analysis displayed an I2 of zero, indicating that the two study populations are similar in terms of population studies (Figure 5) [24]. This sub-analysis showing no statistical significance further points attention to Schwerla et al.’s study swaying the overall results of the meta-analysis [22].

Although this meta-analysis yields significant insights, the study still exhibits several critical limitations. Limitations of the current meta-analysis include a small sample size, high heterogeneity value, concerns about bias, and lack of standardization among the studies included. The lack of standardization can be broken down into differences in anatomical regions treated, inconsistent OMT treatment lengths, and differences in time of pain measurement post-treatment. We believe the high heterogeneity stemmed from multiple reasons, including the general lack of standardization, minimal studies included, and a low number of participants overall. This meta-analysis also acknowledges potential concerns about bias due to the inherent biases within the included studies (Table 1). It is possible that publication bias may have influenced our findings, as our search was limited to studies examining the effects of OMT on pain levels after treatment, assessed via VAS, in patients with joint pain. The funnel plot (Figure 2) indicates that potential bias exists within the studies by Altadonna et al. and Altinbilek et al. Given the previously noted reasons, the statistically significant conclusions should be interpreted with caution. It is important to acknowledge that the clinical applicability and generalizability of the findings may differ from the presented results.

The limitations of this meta-analysis highlight the need for a broader discussion on enhancing OMT research. Overall, there is a limited number of studies on OMT in general as well as the topic of OMT impacting joint pain [3]. It has been found that colleges of osteopathic medicine receive a substantially lower amount of NIH-funded grants in comparison to other institutes [27]. The lack of funding can be a barrier to facilitating research in the field of osteopathy. Due to the lack of research in the field, definitive conclusions can be difficult to make. In the future, we believe it is valuable to conduct multiple randomized controlled trials on each anatomical joint region in substantially sized studies to analyze the difference in pain reduction in patients treated with OMT versus non-OMT. Future studies should ensure the standardization of OMT length of treatment, the specific joint being treated, and the timing of pain measurement after treatment. In addition, larger sample sizes must be used in future studies. With the inclusion of more rigorously conducted studies, appropriate meta-analyses, and systematic reviews can be conducted to make more definitive conclusions. Recently, there has been an increase in evidence-based, government-funded projects, and clinical trials in OMT, and thus the aforementioned suggestions may be a legitimate possibility in the future [1].

Due to the limited amount of research on OMT, the significant results in this meta-analysis can be deemed as important to consider. Overall, our analysis suggests that a definitive conclusion regarding the effectiveness of OMT in significantly reducing joint pain cannot be established. However, the findings suggest that OMT may be a viable option for pain relief in patients with shoulder pain. We believe that additional research is needed to make concrete conclusions regarding the effect of knee and elbow pain.

## Conclusions

In this meta-analysis, we investigated three randomized controlled trials that compared joint pain levels in patients after OMT versus non-OMT. When comparing all studies, it was found that OMT caused a significant reduction in pain for patients utilizing the common effects model, but a significant reduction in pain was not found using the random effects model. Analysis of all three studies and sub-analysis displayed that OMT, which was patient-tailored, was most effective in reducing shoulder pain compared to standard OMT techniques applied to the knee and the elbow. The results of each analysis should be interpreted with caution, as the considerable heterogeneity may influence clinical applicability and limit the ability to draw meaningful interpretations. Nevertheless, given the small sample size and the minimal number of studies, the heterogeneity may have been overestimated. Overall, given the discrepancy in results between the common effects model, random effects model, and high heterogeneity observed, a definitive conclusion regarding the effectiveness of OMT in causing a significant difference in joint pain cannot be drawn. However, the authors propose that OMT may serve as a variable option for pain relief in patients with shoulder pain. Further research is needed to draw definitive conclusions regarding the effectiveness in other joints.
